# Variable Effectiveness: Assessing Methods to Adjust for Covariates in Biomonitoring

**DOI:** 10.1289/ehp.124-A37

**Published:** 2016-02-01

**Authors:** Lindsey Konkel

**Affiliations:** Lindsey Konkel is a New Jersey–based journalist who reports on science, health, and the environment.

Human biomonitoring involves measuring chemical substances, or biomarkers, in people’s blood serum or urine to estimate their exposures.^1^ Investigators typically use statistical adjustments to account for variations in urine dilution or serum lipid levels, because failure to adjust for these factors could result in biased estimates and loss of statistical power.^2^ In this issue of *EHP*, researchers use simulated data to assess how well various standardization techniques, including their own novel approaches, estimate associations between health outcomes and environmental chemicals measured in urine and blood serum.^2^

Better methods for standardizing biomarker measures “should improve our ability to estimate human health risks associated with environmental exposures,” says senior study author Clarice Weinberg, a biostatistician and epidemiologist at the National Institute of Environmental Health Sciences (NIEHS).

**Figure:**
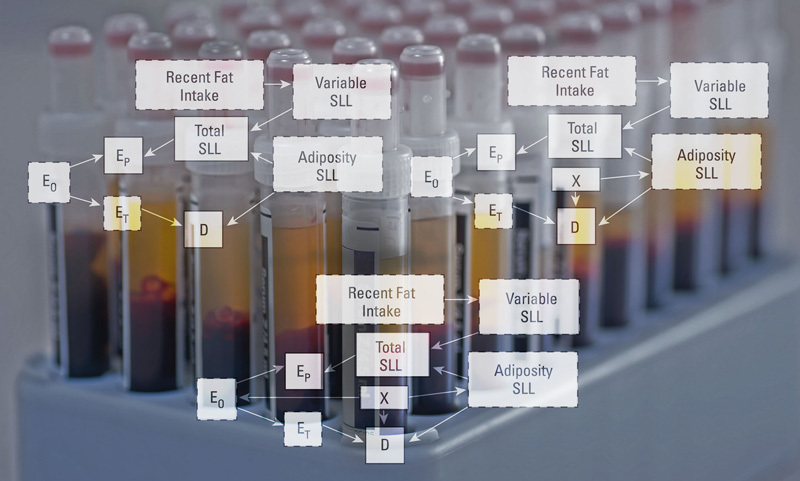
Directed acyclic graphs illustrate the myriad relationships that could exist between exposures, covariates, and disease. These theoretical relationships helped inform new approaches to understanding the potential effects of chemicals measured in biological samples. DAGs: O’Brien et al. (2016)^2^; test tubes: Shutterstock

Urinary diluteness and serum lipid concentrations can vary between individuals as a result of recent water or dietary fat intake. People therefore may have higher or lower concentrations of a chemical in their urine or serum depending on how much they drank or what they ate before providing a sample, variations that may not accurately represent their actual exposures.^2^

Traditionally, when researchers measure chemicals in urine, they divide the concentration of the chemical in urine by the concentration of creatinine, a waste product, in urine. The assumption is that creatinine is excreted at a constant rate both within individuals and among different individuals, such that more concentrated urine has a higher concentration of creatinine. Similarly, when measuring lipophilic chemicals—those that dissolve in fat—researchers divide the amount of chemical in serum by the concentration of lipids in serum.^2^

But some controversy exists over whether this is the best approach to correcting for the variability that occurs among individuals.^3^ Sex, muscle mass, race, and age can influence creatinine levels, while factors unrelated to recent food intake—including sex, age, and body mass index—can affect lipid levels.^3^ If factors that are related to differences in creatinine or serum lipid levels among people are also related to disease risk, traditional adjustment methods may bias associations between the chemical of interest and the related disease.^2^

For the current study, the investigators applied seven standardization approaches to six scenarios in which the concentration of chemical in urine or serum serves as a proxy for the concentration in the disease-relevant target tissue. They used directed acyclic graphs to illustrate possible relationships between actual exposure, concentration of chemical in target tissue, proxy concentration in urine or serum, known and unknown variables including hydration status and creatinine levels (or fat intake and serum lipid levels), and disease as an outcome of exposure.

Along with traditional methods to account for urine dilution and serum lipids, the authors tested a new approach that first models creatinine or serum in relation to other known risk factors. They report that for urine this new approach was better able to reduce measurement error and provide unbiased effect estimates than traditional standardization techniques. For lipophilic chemicals—including most persistent organic pollutants, which are measured in blood—the researchers found that traditional standardization worked better than covariate-adjusted standardization on its own.^2^

The researchers then applied the seven approaches to a real-world situation in which they measured the association between mono-(3-carboxypropyl) phthalate (MCPP) in creatinine and early pregnancy loss in a small group of pregnant women. Some of the approaches indicated a positive association between MCPP and pregnancy loss, while others indicated a negative association. None yielded a statistically significant association. This variability illustrates how the choice of adjustment approach could influence the conclusions of a study.^2^

“This study provides us with good empirical data that in some situations, more complex methods to account for urine dilution may be necessary. As scientists, we need to be thoughtful of how and when to employ these new methods,” says Joe Braun, an epidemiologist at Brown University. Braun was not involved in the current study.

The new methods may prove particularly useful in racially diverse study populations. Whites and Mexican Americans, on average, have lower levels of creatinine in their urine than black Americans.^4^ Low creatinine in urine also may be a predictor of cardiovascular risk.^5^ Braun says researchers looking to study the relationship between environmental exposures and cardiovascular risk, for instance, could benefit from the authors’ methods, which account for the complex relationship between chemical exposures, creatinine, race, and heart disease.
